# Oxidative stress and atherosclerosis: from LDL oxidation and foam cell formation to plaque instability – three decades of evidence and remaining therapeutic challenges

**DOI:** 10.3389/fphys.2026.1968314

**Published:** 2026-10-26

**Authors:** Lili Ji, Haiping Du

**Affiliations:** Department of Cardiovascular Medicine, Yantaishan Hospital, Yantai, China

**Keywords:** antioxidants, atherosclerosis, cardiovascular disease, foam cells, LDL oxidation, matrix metalloproteinases, NADPH oxidase, oxidative stress

## Abstract

Atherosclerosis remains the leading cause of cardiovascular morbidity and mortality worldwide. Over the past three decades, compelling evidence has established oxidative stress as a central pathogenic mechanism in atherogenesis, driving low-density lipoprotein (LDL) oxidation, foam cell formation, vascular inflammation, and ultimately plaque instability. This comprehensive review synthesizes current knowledge on the multifaceted role of reactive oxygen species (ROS) and oxidative stress in atherosclerosis progression. We examine the molecular mechanisms underlying LDL oxidation and its subsequent uptake by macrophage scavenger receptors, leading to foam cell formation. The contribution of oxidative stress to plaque vulnerability through matrix metalloproteinase activation, neovascularization, and necrotic core expansion is critically analyzed. Furthermore, we discuss the translation of this knowledge into therapeutic strategies, including the disappointing outcomes of antioxidant vitamin trials and the emergence of novel targeted approaches such as mitochondria-specific antioxidants and NADPH oxidase inhibitors. Despite significant advances in understanding the pathophysiology, effective anti-atherosclerotic therapies targeting oxidative stress remain elusive. This review identifies persistent gaps in knowledge and outlines future directions for research and therapeutic development. The complex interplay between oxidative stress, inflammation, and lipid metabolism continues to present both challenges and opportunities for cardiovascular medicine.

## 1 Introduction

Cardiovascular diseases (CVDs) remain the leading cause of mortality worldwide, accounting for an estimated 19.8 million deaths in 2022. Atherosclerotic cardiovascular disease, principally ischemic heart disease and stroke, constitutes the major share of this global burden and continues to impose substantial and increasing healthcare demands (Libby et al., 2019; Roth et al., 2020; Collaborators R., Diseases GBoC, 2025). The underlying pathology, atherosclerosis, is a complex lipid-driven chronic inflammatory disease of the arterial wall, characterized by endothelial injury, immune cell infiltration, and fibrofatty plaque buildup across the vascular tree (Sanchis-Gomar et al., 2016; Förstermann et al., 2017; Verboova et al., 2026). Despite widespread clinical adoption of evidence-based therapies including high-intensity statins, PCSK9 inhibitors, antiplatelet regimens, and targeted anti-inflammatory agents residual cardiovascular risk remains unacceptably high, with recurrent ischemic events occurring frequently in treated populations (Singh and Jialal, 2006; Summerhill et al., 2019).

The concept that oxidative stress plays a fundamental role in atherogenesis has evolved considerably over the past three decades. Reactive oxygen species (ROS), once considered merely deleterious byproducts of cellular metabolism, are now recognized as critical signaling molecules that regulate vascular function and pathology (Kattoor et al., 2017). The oxidative modification hypothesis of atherosclerosis, first proposed in the 1980s, posited that the oxidation of low-density lipoprotein (LDL) is an essential step in foam cell formation and atherogenesis (Batty et al., 2022).

This paradigm has since expanded to encompass multiple mechanisms by which oxidative stress promotes all stages of atherosclerosis, from endothelial dysfunction to plaque rupture (Vivekananthan et al., 2003). The pathophysiology of atherosclerosis involves a complex interplay among lipids, inflammatory cells, vascular cells, and mechanical forces. Oxidative stress serves as a unifying mechanism that integrates these diverse factors. ROS promote endothelial activation and dysfunction, modify lipoproteins to enhance their atherogenicity, drive inflammatory cell recruitment and activation, and contribute to the degradation of the extracellular matrix that underlies plaque instability (Loffredo and Carnevale, 2024).

Multiple enzymatic sources contribute to vascular ROS production, including NADPH oxidases, xanthine oxidase, mitochondrial electron transport chains, and myeloperoxidase (Daiber et al., 2017).

Despite the wealth of preclinical evidence supporting the oxidative stress hypothesis of atherosclerosis, translation to effective therapies has proven challenging. Large-scale clinical trials of antioxidant vitamins, including vitamin E, vitamin C, and beta-carotene, have failed to demonstrate consistent benefits for cardiovascular outcomes (An et al., 2022). These disappointing results have prompted a reevaluation of antioxidant strategies and recognition that systemic, non-specific antioxidant approaches may be insufficient or even counterproductive.

This comprehensive perspective piece aims to synthesize three decades of foundational evidence while establishing an updated conceptual framework regarding the role of oxidative stress in atherosclerosis pathogenesis. Rather than providing a purely descriptive summary, we re-evaluate the molecular mechanisms linking redox imbalance to key stage-specific pathological processes highlighting endothelial dysfunction as the initiating interface, the distinct bioactivity of oxysterols and oxidized phospholipids, foam cell formation, and ROS-mediated NLRP3 inflammasome activation driving plaque instability. Furthermore, we critically evaluate targeted therapeutic approaches across the cardiovascular continuum, dissect the reasons for clinical translation gaps, and propose novel future directions for precision redox medicine.

### 1.1 Literature search strategy and methodology

To synthesize three decades of research regarding the role of oxidative stress in atherogenesis, a comprehensive literature search was conducted across PubMed/MEDLINE, Scopus, Web of Science, and Google Scholar databases covering publications from January 1995 through early 2026. The search strategy employed combinations of Medical Subject Headings (MeSH) terms and text words, including: (‘oxidative stress’ OR ‘reactive oxygen species’ OR ‘ROS’) AND (‘atherosclerosis’ OR ‘atherogenesis’ OR ‘endothelial dysfunction’) AND (‘LDL oxidation’ OR ‘oxysterols’ OR ‘foam cell’) AND (‘NLRP3 inflammasome’ OR ‘vascular inflammation’) AND (‘antioxidants’ OR ‘NOX inhibitors’ OR ‘mitochondria-targeted antioxidants’). Inclusion criteria encompassed peer-reviewed original research articles, high-impact clinical trials, and landmark reviews published in English that provided mechanistic insights or translational clinical data. Out of initial search records, studies were selected based on scientific rigor, mechanistic depth, and relevance to the endothelial redox-inflammatory axis.

The primary objective and scientific novelty of this review lie in moving beyond the classical, simplified view of oxidative stress as merely a passive marker of lipid peroxidation. Instead, this review provides an updated integrative framework that highlights endothelial dysfunction as the initiating redox interface, delineates the specific cytotoxic and receptor-mediated effects of bioactive cholesterol oxidation products (oxysterols), maps the stage-specific contributions of cellular ROS sources across the cardiovascular continuum, and critically evaluates the gap between preclinical mechanistic plausibility and demonstrated clinical efficacy of targeted redox therapies.

## 2 Redox pathomechanisms and oxidative stress in atherosclerosis

### 2.1 Sources of reactive oxygen species in the vascular wall

Reactive oxygen species are generated in the vascular wall through multiple enzymatic and non-enzymatic pathways. Understanding the specific sources of ROS is essential for developing targeted therapeutic strategies. NADPH oxidases (Nox) represent the primary enzymatic source of ROS in vascular cells and have emerged as particularly attractive therapeutic targets (Pandey et al., 2012) (**Figure 1**).

**Figure 1:**
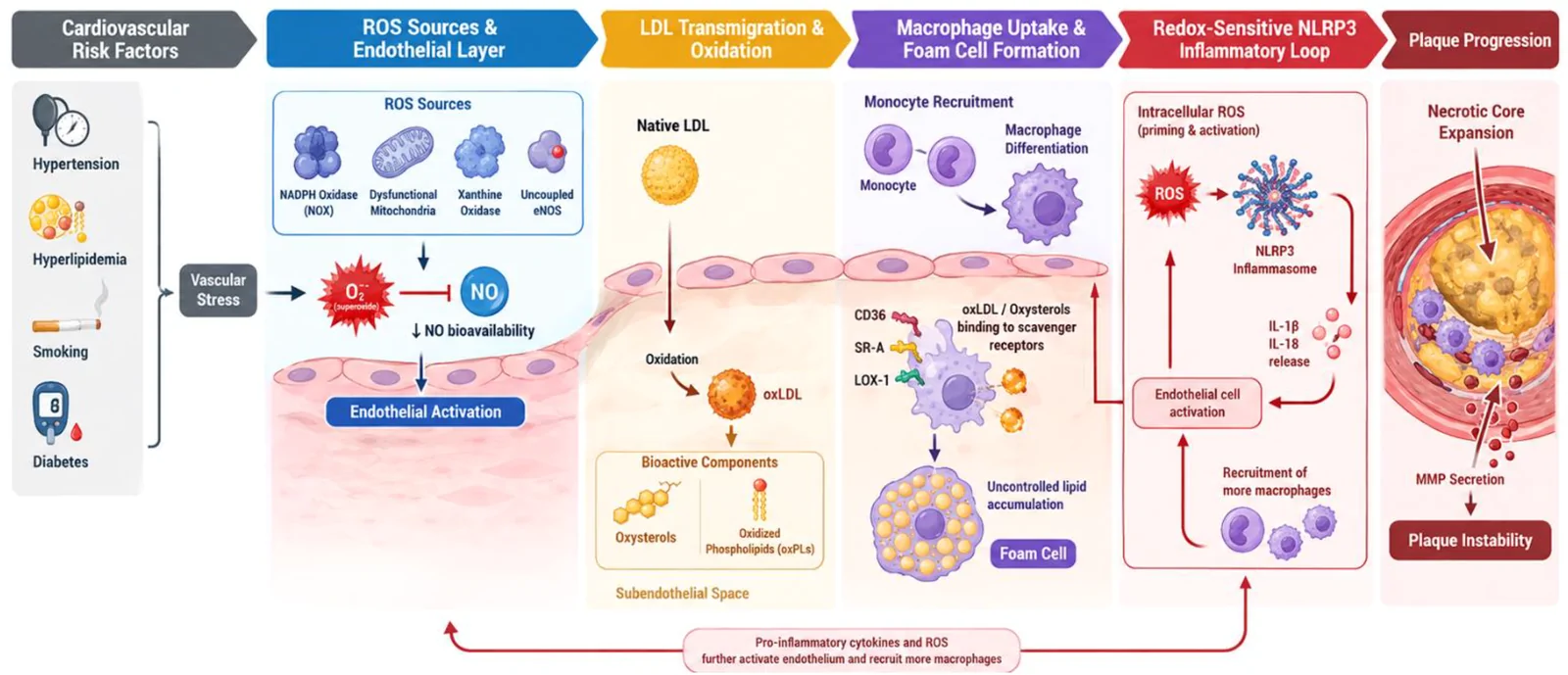
Pathophysiological cascade linking oxidative stress, endothelial dysfunction, and NLRP3 inflammasome activation in atherogenesis. Cardiovascular risk factors induce enzymatic and mitochondrial ROS generation, leading to eNOS uncoupling and reduced nitric oxide (NO) bioavailability. The resulting endothelial activation facilitates subendothelial entry and oxidation of LDL into bioactive oxysterols and oxidized phospholipids. Recruited macrophages internalize these modified lipids via scavenger receptors (CD36, SR-A, LOX-1), resulting in foam cell formation. Intracellular ROS drive NLRP3 inflammasome priming and assembly, triggering IL-1β and IL-18 release. This pro-inflammatory loop perpetuates endothelial activation, leukocyte recruitment, and matrix metalloproteinase (MMP) secretion, ultimately driving necrotic core expansion and plaque instability.

#### 2.1.1 NADPH oxidases

Reactive oxygen species are generated in the vascular wall through multiple enzymatic and non-enzymatic pathways. Understanding the specific sources of ROS is essential for developing targeted therapeutic strategies. Among these, NADPH oxidases (NOX), xanthine oxidase (XO), uncoupled endothelial nitric oxide synthase (eNOS), and myeloperoxidase (MPO) represent the primary enzymatic sources of ROS and RNS in vascular cells (**Figure 2**). The NADPH oxidase family consists of seven isoforms (Nox1–5, Duox1, Duox2) that transfer electrons across biological membranes to reduce oxygen to superoxide (Dikalov et al., 2014). In the vasculature, Nox1, Nox2, Nox4, and Nox5 are the predominant isoforms. Nox2, originally identified in phagocytes, is expressed in endothelial cells and contributes significantly to vascular superoxide production. Notably, studies have shown that Nox2 deficiency attenuates atherosclerosis in experimental models (Barry-Lane et al., 2001; Judkins et al., 2010).

**Figure 2:**
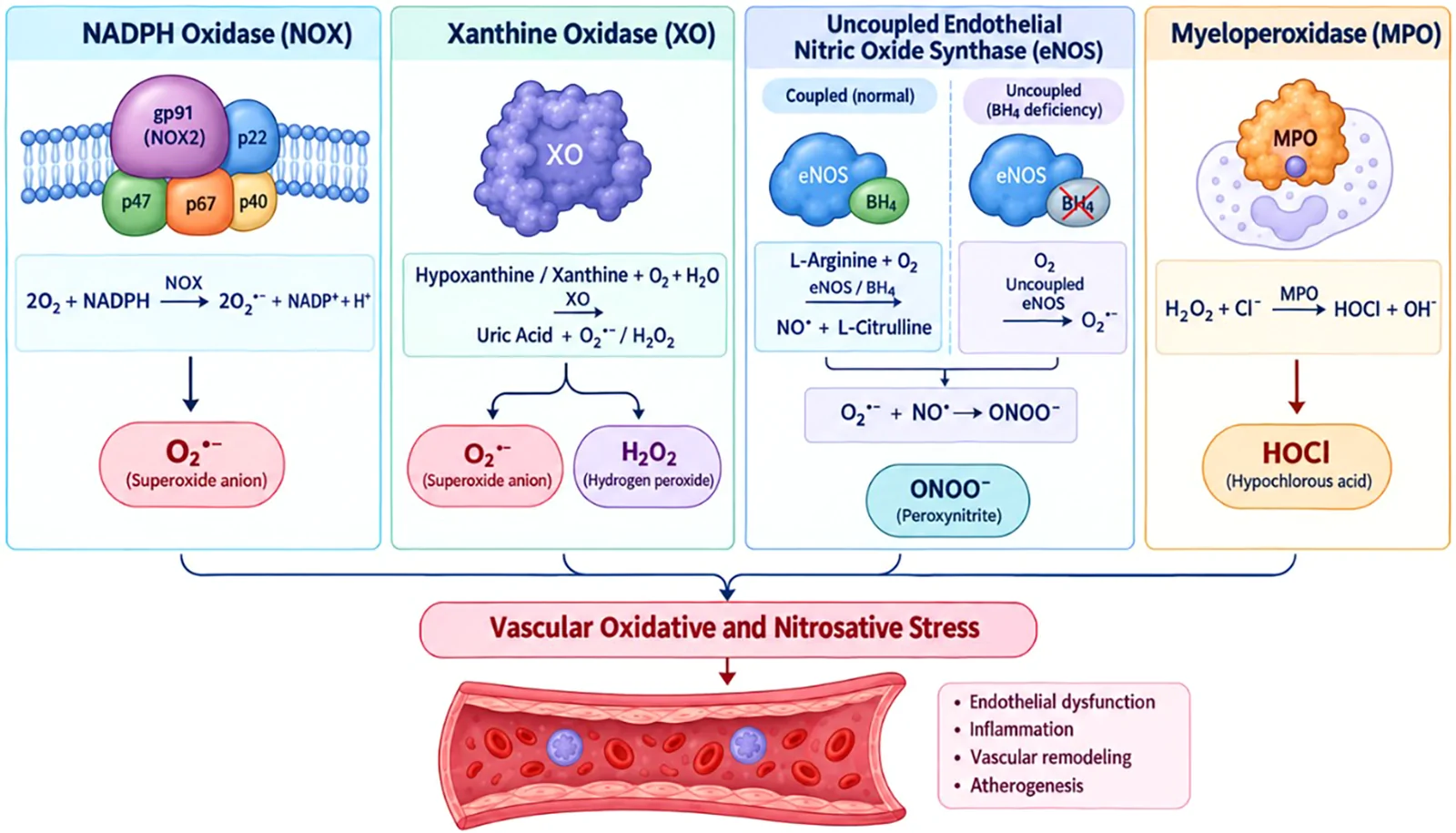
Core enzymatic pathways involved in vascular ROS and RNS generation. Schematic overview of the principal enzymatic sources contributing to oxidative and nitrosative stress during atherogenesis. NADPH oxidase (NOX) transfers electrons across biological membranes, reducing molecular oxygen to superoxide anion. Xanthine oxidase (XO) catalyzes the conversion of hypoxanthine and xanthine to uric acid, generating superoxide anion and hydrogen peroxide as by-products. Under physiological conditions, endothelial nitric oxide synthase (eNOS) produces nitric oxide. However, eNOS uncoupling, commonly associated with tetrahydrobiopterin deficiency, promotes superoxide generation instead. Superoxide rapidly reacts with nitric oxide to form peroxynitrite. Myeloperoxidase (MPO) uses hydrogen peroxide and chloride ions to produce hypochlorous acid. Collectively, these enzymatic pathways promote endothelial dysfunction, vascular inflammation, remodeling, and atherosclerotic progression.

Nox1 is highly expressed in vascular smooth muscle cells (VSMCs) and is upregulated in atherosclerotic lesions (Lassègue et al., 2012). Angiotensin II, a potent pro-atherogenic factor, activates Nox1-mediated ROS production in VSMCs, promoting proliferation, migration, and inflammatory gene expression (Guzik et al., 2006). Nox4, in contrast, appears to have complex effects; while it generates ROS constitutively, it may also exert protective effects through hydrogen peroxide-mediated signaling (Schröder et al., 2012).

Nox5 represents a calcium-dependent isoform that has gained attention in cardiovascular research. Unlike other Nox isoforms, Nox5 contains an N-terminal calcium-binding domain and does not require p47phox or other organizer subunits for activation (BelAiba et al., 2004). Recent studies have demonstrated that Nox5 expression is induced in human atherosclerotic plaques and contributes to endothelial dysfunction and vascular inflammation (Yao et al., 2024). Beyond generating vascular ROS, NADPH oxidases serve as a crucial bridge linking oxidative stress to endothelial dysfunction (ED) and chronic vascular inflammation (Penna and Pagliaro, 2025). High levels of NOX-derived superoxide rapidly react with nitric oxide (NO) to form peroxynitrite, thereby impairing NO bioavailability and shifting the endothelium toward a pro-inflammatory and pro-thrombotic phenotype. Furthermore, NOX-mediated ROS production, alongside mitochondrial dysfunction, acts as a primary upstream signal that triggers both the priming (NF-κB pathway) and activation of the NLRP3 inflammasome within the vascular wall (Penna and Pagliaro, 2025). This leads to the cleavage and release of key pro-inflammatory cytokines, such as IL-1β and IL-18, thereby establishing a self-sustaining loop of endothelial damage, inflammatory cell recruitment, and early atherogenesis (Penna and Pagliaro, 2025).

#### 2.1.2 Mitochondria

The investigation of mitochondrial dysfunction originated in the late 1950s and 1960s, when cellular organelle pathology was first linked to clinical metabolic disorders. Over subsequent decades, the biological understanding of vascular mitochondria shifted from viewing them solely as ATP-generating powerhouses to recognizing them as primary drivers of redox signaling and oxidative stress in vascular cells (Kirkman et al., 2021). Over the last decade, high-resolution multi-omics and genetic models have solidified mitochondrial ROS (mtROS) emission and defective mitophagy as pivotal triggers of endothelial senescence and NLRP3 inflammasome activation across atherogenesis. Today, organelle-targeted antioxidants represent a leading frontier in precision cardiovascular therapeutics (Remex et al., 2026).

Mitochondrial electron transport chain leakage represents a significant source of ROS, particularly under conditions of metabolic stress. Complexes I and III of the respiratory chain are the primary sites of superoxide generation (Brand, 2016). The concept of mitochondrial dysfunction in atherosclerosis has gained considerable traction, with evidence suggesting that mitochondrial ROS (mtROS) contribute to endothelial dysfunction, inflammation, and plaque progression (Chandel, 2015).

Recent studies have demonstrated that macrophage-derived mtROS drive foam cell formation through STAT5 signaling (Boccuni et al., 2025). Mitochondrial superoxide production facilitates macrophage-to-foam cell differentiation by modulating metabolic reprogramming and enhancing chromatin accessibility at STAT5 target genes. This mechanism highlights the importance of mtROS in early atherogenesis and identifies potential therapeutic targets.

#### 2.1.3 Xanthine oxidase and myeloperoxidase

Xanthine oxidase (XO) catalyzes the oxidation of hypoxanthine and xanthine to uric acid, generating superoxide and hydrogen peroxide as byproducts (Kellogg and Fridovich, 1975). XO activity is elevated in patients with cardiovascular disease, and inhibition with allopurinol or febuxostat has been shown to improve endothelial function, although effects on cardiovascular outcomes remain debated (George and Struthers, 2008).

Myeloperoxidase (MPO), a heme peroxidase expressed abundantly in neutrophils and macrophages, generates hypochlorous acid (HOCl) from hydrogen peroxide and chloride (Klebanoff, 2005). MPO-derived oxidants promote atherosclerosis through multiple mechanisms, including modification of LDL, consumption of nitric oxide, and activation of metalloproteinases (Nicholls and Hazen, 2005). MPO is enriched within human atheroma, and elevated plasma MPO levels predict cardiovascular risk independent of traditional risk factors (Brennan et al., 2003).

#### 2.1.4 Uncoupled nitric oxide synthase

Endothelial nitric oxide synthase (eNOS) generates nitric oxide (NO) under normal conditions, contributing to vascular homeostasis. However, when eNOS becomes “uncoupled” due to a deficiency of the cofactor tetrahydrobiopterin (BH4) or the substrate L-arginine, it generates superoxide rather than NO (Forstermann and Münzel, 2006). eNOS uncoupling is observed in various cardiovascular diseases, including atherosclerosis, hypertension, and diabetes (Münzel et al., 2010). Oxidative stress itself can promote eNOS uncoupling by oxidizing BH4, creating a vicious cycle that exacerbates endothelial dysfunction (Landmesser et al., 2003).

### 2.2 Antioxidant defense systems

The vascular wall possesses multiple enzymatic and non-enzymatic antioxidant defense systems that normally maintain redox homeostasis. Superoxide dismutases (SODs) catalyze the dismutation of superoxide to hydrogen peroxide (Fukai and Ushio-Fukai, 2011). Three isoforms exist: cytosolic Cu/Zn-SOD (SOD1), mitochondrial Mn-SOD (SOD2), and extracellular Cu/Zn-SOD (SOD3). SOD3 is particularly important in the vasculature, where it protects against oxidative stress in the extracellular space (Fattman et al., 2003). Together with glutathione peroxidase (GPx), catalase (CAT), and the peroxiredoxin/thioredoxin (Prx/Trx) system, these enzymes cooperatively neutralize reactive oxygen species to preserve endothelial function (**Figure 3**).

**Figure 3:**
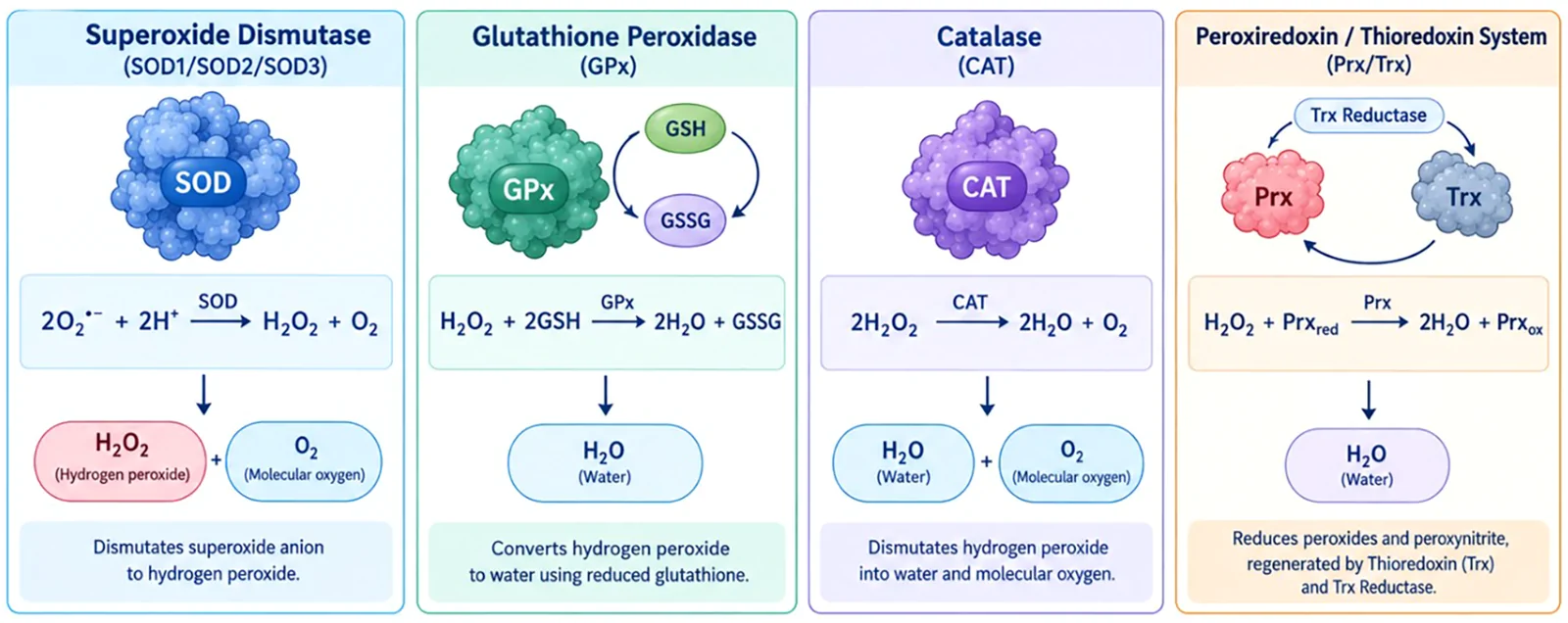
Principal enzymatic antioxidant defense mechanisms in vascular redox homeostasis. Schematic overview of key enzymatic pathways mediating reactive oxygen species (ROS) neutralization in the vascular wall. Superoxide dismutase (SOD1/SOD2/SOD3) catalyzes the dismutation of superoxide anion (O_2_-^-^) into hydrogen peroxide (H_2_O_2_) and molecular oxygen (O_2_). Glutathione peroxidase (GPx) reduces H_2_O_2_ to water (H_2_O) using reduced glutathione (GSH), which is oxidized to glutathione disulfide (GSSG). Catalase (CAT) decomposes excess H_2_O_2_ into water and molecular oxygen. The peroxiredoxin/thioredoxin system (Prx/Trx) reduces peroxides, and oxidized peroxiredoxin is regenerated through the thioredoxin and thioredoxin reductase system. Collectively, these enzymatic pathways help maintain cellular redox balance and protect against vascular oxidative injury.

Catalase and glutathione peroxidases (GPxs) subsequently detoxify hydrogen peroxide to water (Winterbourn, 1995). GPx1, the most abundant GPx isoform, uses reduced glutathione as a cofactor and plays a critical role in preventing oxidative damage (Handy and Loscalzo, 2022). Selenium, an essential component of GPx active sites, influences cardiovascular risk through its effects on antioxidant capacity (Alehagen et al., 2016).

Thioredoxin and peroxiredoxin systems provide additional antioxidant protection by reducing protein thiols and hydrogen peroxide, respectively (Holmgren and Lu, 2010). The thioredoxin-interacting protein (Txnip) negatively regulates thioredoxin function and has been implicated in vascular inflammation and atherosclerosis (Watson et al., 2004).

## 3 LDL oxidation and atherogenicity

### 3.1 Mechanisms of LDL oxidation

The oxidative modification of LDL represents a critical early event in atherogenesis. Native LDL contains lipids susceptible to oxidation, including polyunsaturated fatty acids (PUFAs) in phospholipids and cholesteryl esters (Esterbauer et al., 1990). Oxidation proceeds through multiple pathways involving both free radical-mediated and enzymatic mechanisms.

Free radical-mediated oxidation of LDL occurs through a chain reaction initiated by ROS abstraction of hydrogen atoms from PUFAs, generating lipid radicals (Girotti, 1998). These radicals react with molecular oxygen to form lipid peroxyl radicals, which propagate the chain reaction by abstracting hydrogen from adjacent PUFAs. Lipid hydroperoxides formed through this process decompose into various products, including reactive aldehydes such as malondialdehyde (MDA) and 4-hydroxynonenal (HNE) (Uchida, 2003).

Enzymatic oxidation of LDL may be equally or more important *in vivo*. 12/15-Lipoxygenase (12/15-LOX), expressed in macrophages and endothelial cells, directly oxidizes LDL lipids (Kuhn et al., 2015). MPO generates reactive species that modify both lipid and protein components of LDL (Heinecke et al., 1993). Sphingomyelinase, secreted by activated macrophages, hydrolyzes sphingomyelin in LDL, promoting aggregation and oxidation (Marathe et al., 2003).

### 3.2 Structure and composition of oxidized LDL

Oxidized LDL (oxLDL) is a heterogeneous population of particles with varying degrees of oxidation and biological activity. Minimally modified LDL (mmLDL) represents an early oxidation product that retains apoB-100 recognition by the LDL receptor but acquires pro-inflammatory properties (Berliner et al., 1995). Further oxidation produces fully oxidized LDL, in which apoB-100 lysine residues are modified by reactive aldehydes, preventing recognition by the LDL receptor and enabling uptake by scavenger receptors (Steinbrecher et al., 1990).

The lipid composition of oxLDL includes various bioactive oxidation products. Oxidized phospholipids (oxPLs), particularly those containing fragmented fatty acyl chains, are recognized by specific receptors and contribute to the pro-inflammatory effects of oxLDL (Podrez et al., 2002). Oxysterols, which are oxidation products of cholesterol, accumulate in atherosclerotic plaques and exert cytotoxic and pro-inflammatory effects on vascular cells (Brown and Jessup, 2009).

Unlike unoxidized cholesterol, oxysterols most notably 7-ketocholesterol, 7β-hydroxycholesterol, and 27-hydroxycholesterol represent distinct biologically active species that exert pronounced cytotoxic, pro-inflammatory, and receptor-mediated effects on vascular cells (Poli et al., 2013; Testa et al., 2018). Among these, 7-ketocholesterol is particularly recognized for its marked cytotoxicity, triggering intracellular ROS generation, organelle dysfunction, and macrophage apoptosis (Leonarduzzi et al., 2006). Beyond their direct cytotoxic actions, oxysterols actively modulate scavenger receptor expression; specifically, biologically relevant oxysterol mixtures upregulate CD36 expression in monocytes and macrophages through PKCδ-, ERK1/2-, and PPARγ-dependent signaling pathways, thereby significantly accelerating oxLDL internalization and foam cell formation (Leonarduzzi et al., 2008). Thus, oxysterols serve as critical oxLDL-derived bioactive mediators that directly link cholesterol oxidation to macrophage dysfunction and plaque development.

### 3.3 Biological activities of oxidized LDL

OxLDL exerts multiple pro-atherogenic effects on vascular cells. In endothelial cells, oxLDL induces the expression of adhesion molecules, including vascular cell adhesion molecule-1 (VCAM-1), intercellular adhesion molecule-1 (ICAM-1), and E-selectin, thereby promoting monocyte adhesion and transmigration (Cushing et al., 1990). OxLDL also increases endothelial permeability and induces endothelial apoptosis, compromising the integrity of the endothelial barrier (Cominacini et al., 2001).

In macrophages, oxLDL triggers inflammatory responses through pattern recognition receptors. Toll-like receptor 4 (TLR4) and TLR2 recognize oxLDL components, activating NF-κB and MAPK signaling pathways that drive cytokine production (Miller et al., 2003). The lectin-like oxidized LDL receptor-1 (LOX-1), expressed on endothelial cells, macrophages, and VSMCs, binds oxLDL and mediates internalization, pro-inflammatory signaling, and ROS generation (Kataoka et al., 1999).

OxLDL also exerts chemotactic effects, attracting monocytes and T cells to the arterial wall (Quinn et al., 1987). Furthermore, oxLDL inhibits macrophage migration, potentially trapping these cells within developing lesions (Morel et al., 1984). The pro-thrombotic effects of oxLDL include tissue factor expression, platelet activation, and impairment of fibrinolysis (Penn and Chisolm, 1994).

## 4 Foam cell formation

### 4.1 Macrophage cholesterol uptake via scavenger receptors

Foam cell formation, the hallmark of early atherosclerotic lesions, results from macrophage uptake of modified lipoproteins, particularly oxLDL. Native LDL is not efficiently internalized by macrophages due to the downregulation of LDL receptors by intracellular cholesterol. However, oxLDL is recognized by scavenger receptors that are not regulated by cellular cholesterol content, leading to uncontrolled uptake and cholesterol accumulation (Brown and Goldstein, 1983).

Scavenger receptors represent a structurally diverse superfamily of cell-surface membrane-bound proteins categorized into ten distinct eukaryote families designated Classes A through J, defined by their domain structures, sequence variation, and ligand-binding specificities (Zani et al., 2015). While SRs bind a wide range of endogenous and exogenous ligands involved in pathogen clearance, immune surveillance, and tumor biology, specific subclasses within these groups function as primary drivers of vascular lipid internalization and atherogenesis.

Macrophage internalization of oxidized low-density lipoprotein (oxLDL) is principally mediated by specific scavenger receptors (SRs) acting in concert. Among the Class A scavenger receptor family, which comprises five distinct genes encoding isoforms SR-A1 through SR-A6, the primary gene product driving oxLDL uptake and foam cell formation is scavenger receptor class A member 1 (SR-A1), also designated as macrophage scavenger receptor 1 (MSR1/CD204) (Kunjathoor et al., 2002). Alongside SR-A1, CD36 (SR-B2), a class B scavenger receptor, recognizes oxidized phospholipids and specific oxidized fatty acid modifications on oxLDL subfractions, while LOX-1 (lectin-like oxidized low-density lipoprotein receptor 1/OLR1/SR-E1) plays a complementary role in oxLDL recognition and endothelial-macrophage cross-talk recognize (Febbraio et al., 2000). Genetic knockout models in mice demonstrate that targeted deficiency of either SR-A1 (MSR1) or CD36 (SR-B2) significantly reduces macrophage oxLDL accumulation and attenuates atherosclerotic lesion development (Suzuki et al., 1997).

LOX-1 contributes to foam cell formation, particularly in advanced lesions where it is upregulated on macrophages, endothelial cells, and VSMCs (Mehta et al., 2007). Recent studies have identified additional receptors involved in oxLDL uptake, including SR-PSOX/CXCL16, which functions both as a scavenger receptor and a chemokine (Shimaoka et al., 2000).

### 4.2 Cholesterol esterification and storage

Internalized lipids are transported to late endosomes and lysosomes, where lysosomal acid lipase (LAL) hydrolyzes cholesteryl esters to free cholesterol (Du et al., 1998). Free cholesterol is re-esterified by acyl-CoA:cholesterol acyltransferase-1 (ACAT1) and stored as cytoplasmic lipid droplets, which are characteristic of foam cells (Chang et al., 1993). ACAT1 inhibition prevents cholesterol esterification, increasing free cholesterol levels to concentrations that are toxic to cells (Accad et al., 2000).

The balance between cholesterol influx and efflux determines foam cell formation. ATP-binding cassette transporter A1 (ABCA1) and ABCG1 mediate cholesterol efflux from macrophages to apolipoprotein A-I and mature high-density lipoprotein (HDL), respectively (Wang and Oram, 2002). Impaired cholesterol efflux, due to reduced ABCA1/ABCG1 expression or dysfunctional HDL, promotes foam cell accumulation (Wang et al., 2007).

### 4.3 Cellular consequences of foam cell formation

Intracellular lipid accumulation initiates a pathologic cascade that transitions functional macrophages into dysfunctional foam cells. Following scavenger receptor-mediated oxLDL uptake and lysosomal hydrolysis, persistent free cholesterol overload surpasses the re-esterification capacity of ACAT1 and the efflux mechanisms of ABCA1/ABCG1. This unesterified cholesterol accumulates within organelle membranes, directly triggering prolonged endoplasmic reticulum (ER) stress via the unfolded protein response, mitochondrial membrane dysfunction, and inflammatory activation (Glass and Witztum, 2001; Tabas, 2010).

Foam cells are active drivers of atherogenesis rather than passive lipid storage depots. Cholesterol-overloaded macrophages exhibit heightened inflammatory signaling, releasing key cytokines, chemokines, and matrix metalloproteinases that accelerate lesion destabilization (Araujo et al., 2025).

Furthermore, foam cell formation is tightly coupled with metabolic reprogramming. OxLDL internalization induces a metabolic shift toward aerobic glycolysis (the Warburg effect), providing critical biosynthetic precursors to sustain pro-inflammatory responses (Freemerman et al., 2014). Pyruvate kinase M2 (PKM2), a rate-limiting glycolytic enzyme, plays a pivotal role in mediating this oxLDL-induced glycolytic shift and exacerbating vascular inflammation (Kumar et al., 2020).

Chronic organellar stress ultimately pushes foam cells toward apoptotic cell death (Tabas, 2009). In early atherosclerotic lesions, apoptotic foam cells are efficiently cleared by neighboring phagocytes via efferocytosis. However, in advanced plaques, persistent oxidative stress and oxLDL exposure impair efferocytosis recognition signals (Schrijvers et al., 2005; Thorp and Tabas, 2009). Uncleared apoptotic foam cells subsequently undergo secondary necrosis, releasing intracellular lipids, oxidized debris, and pro-inflammatory danger signals that aggregate to drive acellular necrotic core expansion (Yurdagul et al., 2018).

## 5 Oxidative stress and plaque instability

### 5.1 Characteristics of vulnerable plaques

Acute cardiovascular events typically result from the rupture or erosion of atherosclerotic plaques, rather than progressive luminal narrowing (Falk et al., 1995). Vulnerable plaques exhibit specific morphological features: a large lipid-rich necrotic core (>40% of plaque volume), a thin fibrous cap (<65 μm), active inflammation with abundant macrophages, and neovascularization (Virmani et al., 2007). Oxidative stress contributes to each of these features, promoting plaque vulnerability and increasing the risk of rupture. As illustrated in **Figure 4**, advanced vulnerable plaques exhibit high expression of specific enzymatic and receptor biomarkers (e.g., MMPs, LOX-1, MPO). Beyond driving cap erosion and thrombosis, these plaque-specific biomarkers represent valuable molecular targets for non-invasive diagnostic monitoring as well as precision prodrug targeting to promote lesion regression in complex vascular networks, including the brain.

**Figure 4:**
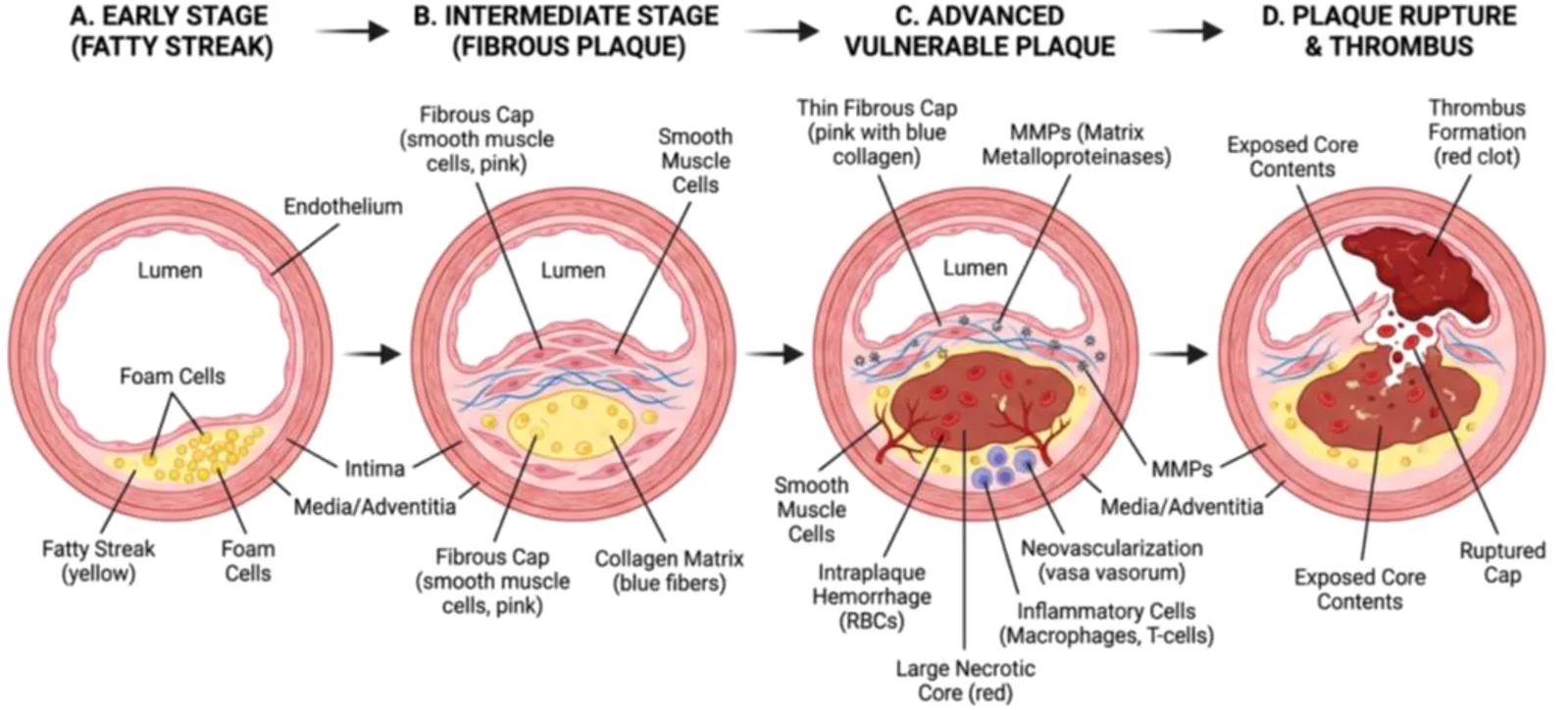
Stages of atherosclerotic plaque progression, rupture, and targeted clinical applications. **(A)** Early fatty streak formation with foam cells. **(B)** Intermediate fibrous plaque with smooth muscle cell migration. **(C)** Advanced vulnerable plaque showing necrotic core expansion, thin cap erosion by matrix metalloproteinases (MMPs), and intraplaque hemorrhage. **(D)** Plaque rupture exposing thrombogenic material. Translational Relevance: Plaque-overexpressed biomarkers (e.g., MMP-2/9, LOX-1, MPO) serve as diagnostic tools and molecular targets for site-specific prodrug/nanomedicine delivery to resolve vulnerable lesions in complex vascular beds like the brain.

The fibrous cap provides structural integrity to atherosclerotic plaques, preventing contact between thrombogenic plaque contents and circulating blood. Matrix metalloproteinases (MMPs), a family of zinc-dependent proteases, degrade extracellular matrix components, weakening the fibrous cap and promoting plaque rupture (Newby, 2006).

Oxidative stress activates MMPs through multiple mechanisms. ROS directly activate MMPs by oxidizing cysteine residues in their pro-domain, thereby relieving autoinhibition (Okamoto et al., 2001). Superoxide and hydrogen peroxide activate pro-MMP-2 and pro-MMP-9 through this mechanism (Rajagopalan et al., 1996). Additionally, ROS increase MMP expression through the activation of transcription factors, including NF-κB and AP-1 (Galis et al., 1994).

MMP-1 (interstitial collagenase), MMP-8 (neutrophil collagenase), and MMP-13 (collagenase-3) cleave fibrillar collagens, the major structural components of the fibrous cap (Lijnen, 2003). MMP-9 (gelatinase B) degrades denatured collagen and basement membrane components. Studies of human coronary plaques have demonstrated co-localization of MMP-1 with regions of high circumferential stress, suggesting that mechanical stress and proteolytic degradation collaborate to promote plaque rupture (Lee et al., 1996).

### 5.2 Necrotic core expansion and cholesterol crystals

The necrotic core develops from macrophage death and the accumulation of cellular debris, cholesterol crystals, and oxidized lipids. Oxidative stress contributes to necrotic core expansion by promoting macrophage apoptosis and impairing efferocytosis (Seimon et al., 2009). Cholesterol crystals, formed from excess free cholesterol, are potent activators of the NLRP3 inflammasome, driving IL-1β production and inflammation (Duewell et al., 2010). Beyond physical activation by cholesterol crystals, NLRP3 inflammasome activity is also modulated by cellular redox imbalance. Mitochondrial dysfunction and enzymatic ROS sources, including xanthine oxidase and NADPH oxidases, can promote NLRP3 priming and assembly, thereby amplifying inflammatory signaling in the vascular wall (Zhou et al., 2011; Abais et al., 2015).

Hypoxic conditions within advanced plaques induce the expression of hypoxia-inducible factors (HIFs), which regulate adaptation to low oxygen. HIF-1α stabilization promotes the expression of genes involved in glycolysis, angiogenesis, and iron metabolism (Sluimer et al., 2008). Recent studies have shown that high expression of heme oxygenase-1 (HO-1), a HIF-1α target gene, under hypoxic conditions drives ferroptosis in macrophage-derived foam cells, exacerbating plaque instability (Guo et al., 2024).

### 5.3 Intraplaque hemorrhage and neovascularization

Advanced plaques develop neovessels originating from the adventitial vasa vasorum that penetrate into the intima. These neovessels are structurally abnormal, with defective endothelial junctions that permit the leakage of plasma components and erythrocytes into the plaque (Moreno et al., 2004). Intraplaque hemorrhage delivers erythrocytes rich in cholesterol and iron, promoting inflammation and oxidative stress (Kolodgie et al., 2003).

Heme and iron released from erythrocytes generate ROS through Fenton chemistry, propagating oxidative stress within the plaque (Winterbourn, 1995). Iron accumulation in macrophages promotes foam cell formation and oxidative stress. Erythrocyte-derived phospholipids contribute to the lipid pool, while hemoglobin scavenging by macrophages induces HO-1 expression, which has complex effects on plaque stability (Takaya et al., 2006).

### 5.4 Smooth muscle cell phenotype and fibrous cap integrity

Vascular smooth muscle cells (VSMCs) in advanced lesions can transdifferentiate into a macrophage-like phenotype, characterized by the downregulation of contractile markers and the upregulation of macrophage markers, including CD68 and Mac-2 (Shankman et al., 2015). This phenotypic transition is driven by inflammatory cytokines, oxLDL uptake, and oxidative stress. VSMC-derived foam cells contribute to the necrotic core and may compromise fibrous cap integrity (Feil et al., 2014).

Oxidative stress inhibits VSMC proliferation and collagen synthesis while promoting apoptosis. These effects, combined with MMP-mediated matrix degradation, contribute to fibrous cap thinning. The balance between VSMC-mediated repair and inflammatory destruction determines plaque stability (Bennett et al., 2016).

## 6 Clinical evidence linking oxidative stress to atherosclerosis

### 6.1 Biomarkers of oxidative stress

During the late twentieth century, increasing clinical interest in oxidative stress prompted the measurement of circulating biomarkers to estimate systemic redox imbalance. Early clinical investigations frequently relied on global downstream indices of lipid peroxidation, particularly plasma malondialdehyde (MDA) and thiobarbituric acid-reactive substances (TBARS), although the analytical specificity of conventional TBARS-based assays is limited (Del Rio et al., 2005; Frijhoff et al., 2015). However, the clinical translational utility of classical lipid-peroxidation assays has been limited by analytical non-specificity, susceptibility to ex vivo artifact formation, and the fact that circulating concentrations reflect both biomarker generation and clearance. More recently, biomarker development has shifted toward better-characterized oxidative and inflammatory pathways, including oxidized LDL-related measures, myeloperoxidase (MPO), and mass spectrometry–based urinary F2-isoprostanes. These markers are being evaluated as complementary tools for mechanistic phenotyping and risk assessment in acute coronary syndromes and atherosclerotic cardiovascular disease (Frijhoff et al., 2015).

Numerous studies have demonstrated associations between biomarkers of oxidative stress and cardiovascular disease. Plasma levels of oxidized LDL, measured by various assays, correlate with coronary artery disease severity and predict cardiovascular events independent of traditional risk factors (Itabe, 2012). MDA, a product of lipid peroxidation, is elevated in patients with cardiovascular disease, although its measurement is subject to technical limitations (Tsikas, 2017).

Myeloperoxidase has emerged as a particularly promising biomarker. Elevated plasma MPO levels predict the risk of cardiovascular events in patients with acute coronary syndromes and stable coronary artery disease (Heslop et al., 2010). However, the utility of MPO as a clinical biomarker remains limited by issues regarding assay standardization and specificity.

Antioxidant enzyme activities have also been investigated as markers of oxidative stress. Reduced activities of SOD, catalase, and GPx have been reported in patients with coronary artery disease (Gupta et al., 2009). However, the interpretation of these findings is complicated by potential adaptive responses to chronic oxidative stress.

### 6.2 Genetic evidence

Genetic studies provide additional support for the role of oxidative stress in atherosclerosis. Polymorphisms in genes encoding antioxidant enzymes have been associated with cardiovascular risk. The Ala16Val polymorphism in manganese SOD (SOD2) affects enzyme localization and activity, with the Val variant associated with increased cardiovascular risk in some studies (Kašparová et al., 2005). Haplogroups of mitochondrial DNA encoding different variants of respiratory chain components may influence ROS production and cardiovascular risk (Stocker and Keaney, 2004).

## 7 Therapeutic challenges and strategies

### 7.1 Lessons from antioxidant vitamin trials

The translation of oxidative stress research into effective therapies has proven remarkably challenging. Large-scale randomized controlled trials of antioxidant vitamins, including vitamin E, vitamin C, and beta-carotene, have generally failed to demonstrate cardiovascular benefits and, in some cases, have suggested harm (Clarke and Armitage, 2002).

The HOPE and HOPE-TOO trials investigated vitamin E supplementation (400 IU/day) in patients at high cardiovascular risk (Yusuf et al., 2000). No reduction in cardiovascular events was observed, and there was a suggestion of increased heart failure risk. The Women’s Health Study evaluated vitamin E in primary prevention among 39,876 women, finding no effect on major cardiovascular events (Lee et al., 2005). A meta-analysis of randomized trials concluded that vitamin E supplementation does not reduce cardiovascular mortality or events (Jha et al., 1995).

Several factors may explain these disappointing results. Systemic antioxidant supplementation may not effectively reduce oxidative stress at critical cellular compartments. The timing of intervention may also be important; antioxidant supplementation initiated after established atherosclerosis may be too late to modify disease progression. Furthermore, some ROS serve important physiological signaling functions, and non-specific antioxidant suppression may have detrimental effects (Steinberg, 2006).

### 7.2 Targeting specific ROS sources

Recognition of the limitations of systemic antioxidant approaches has stimulated interest in targeted therapies directed at specific enzymatic sources of ROS. NADPH oxidase inhibitors represent a particularly promising therapeutic strategy. Small molecule inhibitors of Nox2 and other isoforms have been developed and show promise in preclinical models (Griendling et al., 2016).

Xanthine oxidase inhibitors, including allopurinol and febuxostat, have been extensively studied. While these drugs effectively reduce uric acid levels and may improve endothelial function, the relative contributions of the anti-atherogenic effects of uric acid reduction versus the antioxidant properties of XO inhibition remain debated (Korsmo et al., 2024).

### 7.3 Mitochondria-targeted antioxidants

The recognition that mitochondria are both sources and targets of ROS has led to the development of mitochondria-targeted antioxidants. MitoQ, a ubiquinone derivative conjugated to a lipophilic triphenylphosphonium cation, accumulates in mitochondria based on membrane potential and acts as an antioxidant (Smith et al., 2003). Preclinical studies have shown protective effects in various cardiovascular models, and clinical trials have demonstrated safety and potential benefits in Parkinson’s disease and hepatitis C.

Other mitochondria-targeted antioxidants include SS-31 (Elamipretide), a peptide that targets cardiolipin and preserves mitochondrial function (Sabbah et al., 2016). Clinical trials of SS-31 in heart failure and mitochondrial diseases are ongoing. These targeted approaches may avoid the pitfalls of systemic antioxidant supplementation while effectively reducing pathological mtROS production.

### 7.4 Anti-inflammatory approaches

Given the intimate relationship between oxidative stress and inflammation, anti-inflammatory strategies may indirectly reduce oxidative stress in atherosclerosis. The CANTOS trial demonstrated that canakinumab, an IL-1β neutralizing antibody, reduced cardiovascular events in patients with residual inflammatory risk (Ridker et al., 2017a). This landmark study established inflammation as a therapeutic target in atherosclerosis.

Colchicine, an inexpensive anti-inflammatory agent that inhibits microtubule polymerization and NLRP3 inflammasome activation, has shown promise in cardiovascular disease. The COLCOT trial demonstrated reduced cardiovascular events with low-dose colchicine following myocardial infarction (Tardif et al., 2019). The LoDoCo2 trial extended these findings to chronic coronary disease. The anti-inflammatory effects of colchicine likely include the reduction of oxidative stress through multiple mechanisms.

### 7.5 Statins and pleiotropic effects

Statins, the cornerstone of atherosclerosis prevention, exert pleiotropic effects beyond LDL lowering that include antioxidant properties. Statins reduce NADPH oxidase expression and activity, increase eNOS expression, and improve antioxidant enzyme activities (Wassmann et al., 2003). These effects contribute to the benefits of statins that cannot be fully explained by LDL reduction alone.

The anti-inflammatory and antioxidant effects of statins may be particularly important in high-risk patients. High-intensity statin therapy reduces markers of oxidative stress and improves endothelial function independent of LDL lowering (Endo, 1992). A comprehensive synthesis and comparative analysis of these translational antioxidant and anti-inflammatory strategies—highlighting their molecular targets, experimental efficacy, clinical outcomes, and limitations—is presented in **Table 1**.

**Table 1:** Overview of antioxidant, redox-targeted, and anti-inflammatory therapeutic strategies in atherosclerosis: molecular targets, mechanisms, clinical evidence, limitations, and development status.

Therapeutic strategy	Molecular target/ROS source	Proposed mechanism	Experimental evidence	Available clinical evidence	Principal limitations	Stage of development	Ref.
Antioxidant vitamins Vitamin E, vitamin C,	Systemic and lipid-associated oxidative stress; lipid peroxyl radicals	Broad reduction of lipid oxidation and radical propagation	Vitamin E reduced progression of established atherosclerotic lesions in LDLR-deficient mice in an experimental model	HOPE and WHS showed no significant reduction in major cardiovascular events with vitamin E supplementation	Poor lesion/cellular targeting; possible interference with physiological redox signaling; inconsistent translation from animal models	Not supported for routine primary or secondary CVD prevention	(Yusuf et al., 2000; Cyrus et al., 2003; Averill et al., 2009)
NADPH oxidase inhibitors GKT137831/setanaxib	Mainly NOX1 and NOX4; vascular ROS production	Reduces vascular superoxide and hydrogen peroxide production through NOX1/4 inhibition	GKT137831 produced dose-dependent reno- and atheroprotection in diabetic mice with established vascular disease	Early phase clinical studies have evaluated safety and efficacy mainly in diabetic kidney disease; cardiovascular efficacy remains unproven	Incomplete isoform selectivity; uncertain long-term safety; possible interference with physiological redox signaling	Preclinical to early clinical development; no established CVD indication	(Gray et al., 2017; De Livera et al., 2020)
Xanthine oxidase inhibitors Allopurinol, febuxostat	Xanthine oxidase; purine-derived superoxide	Inhibits purine oxidation and reduces uric acid and potentially XO-derived ROS	High-dose allopurinol improved endothelial function and reduced vascular oxidative stress in patients with endothelial dysfunction	ALL-HEART showed no reduction in cardiovascular death, nonfatal MI, or nonfatal stroke with allopurinol in ischemic heart disease	Uric-acid lowering and ROS inhibition cannot be separated; febuxostat has cardiovascular-safety concerns	FDA-approved for gout; not established for CVD prevention	(George et al., 2006; Mackenzie et al., 2022)
Mitochondria-targeted antioxidants MitoQ, SS-31/elamipretide	Mitochondrial ROS and cardiolipin-associated oxidative injury	Mitochondrial accumulation reduces mtROS and may preserve mitochondrial membrane and cardiolipin integrity	Mitochondria-targeted antioxidants reduced mitochondrial oxidative stress and vascular dysfunction in experimental models	MitoQ improved endothelial function and reduced aortic stiffness in a small randomized study of older adults; cardiovascular outcome benefit remains unproven	Dose- and tissue-dependent effects; uncertain long-term efficacy; limited cardiovascular outcome data	Investigational; early clinical development	(Szeto and Birk, 2014; Rossman et al., 2018)
Statins Atorvastatin, rosuvastatin	HMG-CoA reductase; secondary effects on Rac1/NOX, eNOS coupling, and vascular inflammation	LDL-C lowering with additional anti-inflammatory and vascular redox effects	Statins reduced vascular superoxide formation and improved endothelial nitric oxide-dependent vasodilation in experimental and translational studies	JUPITER demonstrated a significant reduction in major cardiovascular events with rosuvastatin	Myopathy, drug interactions, and modest risk of new-onset diabetes; residual inflammatory risk remains	FDA-approved; first-line standard of care for ASCVD risk reduction	(Wagner et al., 2000; Ridker et al., 2008)
Canakinumab	IL-1β downstream of NLRP3 inflammasome activation	Neutralizes IL-1β and suppresses vascular inflammation and inflammation-associated oxidative signaling	Reduced macrophage activation and inflammatory vascular responses in experimental models	CANTOS showed reduced recurrent cardiovascular events with canakinumab independently of LDL-C lowering	Increased risk of serious and fatal infections; high cost; no broad regulatory approval for CVD prevention	Clinical proof-of-concept; not approved for routine CVD prevention	(Ridker et al., 2017a; Ridker et al., 2017b)
Colchicine	Microtubules, NLRP3 inflammasome assembly, and neutrophil activation	Inhibits microtubule-dependent inflammatory signaling, inflammasome activation, and neutrophil responses	Reduced vascular inflammatory responses and leukocyte-mediated atherogenic signaling in experimental models	COLCOT and LoDoCo2 showed reductions in composite ischemic cardiovascular outcomes in selected post-MI and chronic coronary disease populations	Gastrointestinal toxicity, narrow therapeutic window, myotoxicity, and drug interactions	Established adjunctive therapy in selected CAD populations	(Tardif et al., 2019; Nidorf et al., 2020)

## 8 Discussion

Over the past three decades, understanding of oxidative stress in atherogenesis has evolved from a relatively simplified model centered on oxidative modification of LDL to a more complex view involving spatially compartmentalized redox signaling, inflammation, and cell-specific oxidative pathways. During the 1990s, the oxidative modification hypothesis fostered the expectation that broadly acting antioxidant strategies, including vitamins E and C, might attenuate LDL oxidation and reduce atherosclerotic disease; however, large clinical trials of nonspecific antioxidant supplementation were largely disappointing (Steinberg, 2009). Indeed, the unequivocal failure of large-scale clinical vitamin trials forced a fundamental scientific pivot, exposing the limitations of global ROS clearance and highlighting the essential physiological roles of ROS in cellular homeostasis and vascular signaling.

As understanding of vascular redox biology advanced, attention shifted toward discrete cellular sources of oxidative stress. Enzymatic ROS generators, particularly NADPH oxidases (NOX) and uncoupled endothelial nitric oxide synthase (eNOS), are now recognized as important contributors to endothelial dysfunction, vascular inflammation, and atherogenesis. More recently, organelle-specific studies have highlighted mitochondrial dysfunction as both a consequence and an amplifier of vascular injury. Excess mitochondrial ROS (mtROS) and defective mitophagy contribute to endothelial senescence, inflammatory signaling, and the progression of atherosclerosis (Förstermann et al., 2017).

Parallel to these pathophysiological insights, therapeutic strategies have experienced a clear evolutionary dynamic. The therapeutic emphasis in atherosclerotic cardiovascular disease has increasingly shifted from broad, non-specific antioxidant supplementation toward pathway-informed anti-inflammatory and redox-modulating strategies. Among clinically evaluated approaches, low-dose colchicine and IL-1β blockade with canakinumab have provided proof-of-concept that targeting the NLRP3–IL-1β inflammatory axis can reduce residual inflammatory cardiovascular risk. Nevertheless, substantial translational challenges remain, including heterogeneity in inflammatory phenotypes, uncertainty regarding optimal patient selection, and the need to preserve protective host-defense and physiological signaling pathways (Nelson et al., 2023).

Looking forward, advancing precision redox medicine in cardiovascular disease will require integrating compartmentalized redox dynamics with immunometabolism, trained immunity, and single-cell multi-omics profiling. Tailoring targeted redox therapies to specific disease stages and individual patient biomarker profiles represents the most viable pathway to bridge the longstanding gap between preclinical mechanistic plausibility and clinical therapeutic efficacy.

Translating molecular targets into verified clinical modalities requires rigorous validation within global registries such as ClinicalTrials.gov. Although emerging biomarkers like soluble LOX-1 (sLOX-1) offer strong mechanistic rationale for monitoring intraplaque oxidative dynamics, their clinical acceptance remains limited by assay standardization and variable predictive power across diverse trial cohorts (Barreto et al., 2021). This diagnostic lag is mirrored in therapeutic translation; for instance, targeting LOX-1 via humanized monoclonal antibodies (e.g., MEDI6570) has yielded inconclusive clinical outcomes, underscoring the complexity of neutralizing multi-ligand scavenger pathways. Conversely, the clinical success of anti-PCSK9 approaches including monoclonal antibodies and siRNA platforms demonstrates the efficacy of targeted molecular interception over traditional statins. Despite higher cost barriers restricting widespread first-line deployment, anti-PCSK9 therapeutics represent a critical disease-modifying alternative for the estimated 20% of cardiovascular patients with documented statin intolerance, illustrating the necessity of aligning biomarker discovery with stratified patient selection in late-phase clinical evaluation (Pokhrel et al., 2024; Sánchez-León et al., 2024).

## 9 Conclusion

Three decades of extensive biomedical research have firmly established oxidative stress as a central, compartmentalized mechanism linking cardiovascular risk factors to all stages of atherosclerosis pathogenesis. As synthesized in this comprehensive perspective, vascular redox biology underwent a fundamental paradigm shift—moving away from the early, oversimplified view of global lipid oxidation toward a highly nuanced understanding of organelle-specific ROS signaling, uncoupled enzymatic pathways, and ROS-driven NLRP3 inflammasome activation.

The clinical failure of early non-specific antioxidant vitamin trials does not invalidate the oxidative stress hypothesis; rather, it underscores the critical necessity for targeted, site-specific therapeutic strategies. Emerging approaches—including mitochondria-targeted antioxidants, selective NADPH oxidase inhibitors, and dual redox-inflammatory modulators—offer genuine promise for overcoming past translational barriers.

Bridging the gap between preclinical mechanistic plausibility and clinical therapeutic success requires transitioning toward precision redox medicine. By integrating compartmentalized redox therapeutics with validated biomarkers, immunometabolic profiling, and stage-specific patient stratification, the next era of cardiovascular research holds the potential to convert hard-won mechanistic insights into effective clinical interventions against ASCVD.
